# Supplementary material for: The Reproductive Biology of Puye (Galaxias maculatus) under Experimental Culture
Source: Animals (Basel). 2024 Jan 19;14(2):320. doi: 10.3390/ani14020320 (PMC10812412; doi:10.3390/ani14020320)
Supplement: Supplementary file 1 [file animals-14-00320-s001.zip › animals-2721781-supplementary.pdf]

animals

MDPI

Supplementary material

Supplementary Table S1. Macroscopic evaluation of sexual maturity in *Galaxias maculatus* for a population held in captivity. Adapted from Mardones et al., 2008.

State	External Characteristics		
Immaturity	State used interchangeably for males and females. It corresponds to specimens that do not show external signs of maturity. The fish are characterised by a completely silver abdominal wall. It is not possible to differentiate males from females.	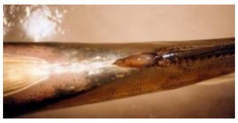	Female
		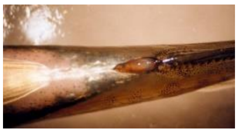	Male
Initial maturity	*Females:* Very small yellowish-looking oocytes are observed, which can be seen through the abdominal wall near the urogenital pore. Generally, the anterior abdominal region remains slightly silvery.	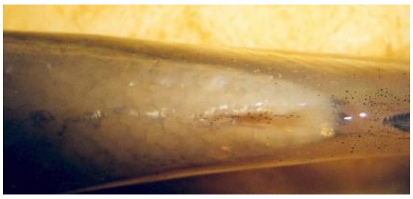	
	Males: It is possible to identify a whitish internal testicle and a silver abdominal wall.	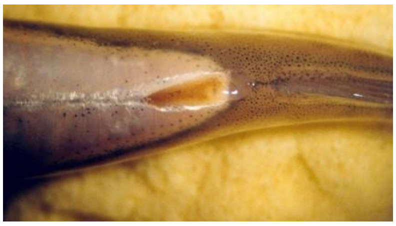	
Advanced maturity	*Females:* The abdominal region is hard to the touch. Compared to the previous state, it presents a greater bulge due to the presence of growing oocytes located in rows in the posterior region of the gonad. The appearance of the oocytes is whitish.	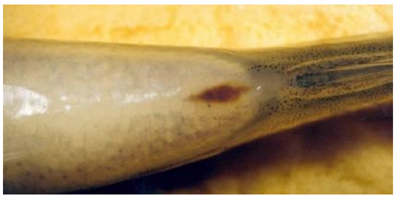	
	Males: The individuals show a significant bulge in the abdominal region. The whitish-looking testicles develop towards the lateral margins of the genital pore.	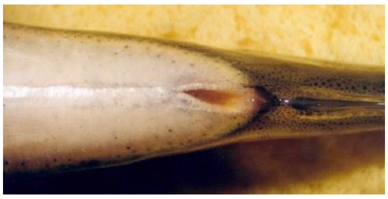	

Maximum maturity	*Females:* Females have a distended abdomen and a very dilated genital pore. Translucent-looking oocytes are found in the abdominal cavity and can be easily released by gentle abdominal massage.	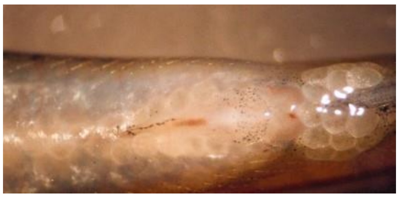
	Males: Males have very developed testicles and, therefore, a very bulging abdomen. Semen can be easily extracted by applying light pressure to the abdominal region. The semen is characterised by its white and very fluid appearance.	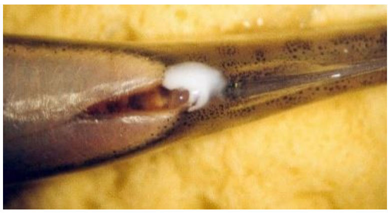
Resorption	*Females:* The abdomen is underdeveloped and thinner in the genital pore region. Portions heterogeneous in shape, texture and colour are observed in the abdominal region. The transparency of the abdominal wall is regularly lost.	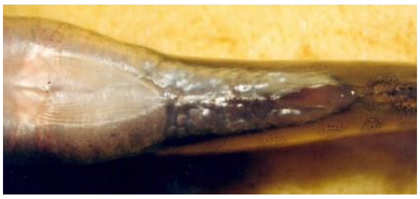
	Males: State of difficult macroscopic identification in males, as they can be confused with specimens in initial maturity. Despite a well-developed and whitish gonad being observed because of transparency, there is no release of mature gametes.	
